# THE GENETIC BASIS OF A COMPLEX FUNCTIONAL SYSTEM

**DOI:** 10.1111/j.1558-5646.2012.01688.x

**Published:** 2012-05-28

**Authors:** Nicholas F Parnell, C Darrin Hulsey, J Todd Streelman

**Affiliations:** 1 School of Biology, Institute of Bioengineering and Bioscience, Georgia Institute of TechnologyAtlanta, Georgia 30332; 3 Department of Ecology and Evolutionary Biology, University of TennesseeKnoxville, Tennessee 37996

**Keywords:** Cichlid, epistasis, genomic “hotspots”, jaw, many-to-one mapping, QTL, transgressive segregation

## Abstract

The relationship between form and function can have profound effects on evolutionary dynamics and such effects may differ for simple versus complex systems. In particular, functions produced by multiple structural configurations (many-to-one mapping, MTOM) may dampen constituent trade-offs and promote diversification. Unfortunately, we lack information about the genetic architecture of MTOM functional systems. The skulls of teleost fishes contain both simple (lower jaw levers) as well as more complex (jaws modeled as 4-bar linkages) functional systems within the same craniofacial unit. We examined the mapping of form to function and the genetic basis of these systems by identifying quantitative trait loci (QTL) in hybrids of two Lake Malawi cichlid species. Hybrid individuals exhibited novelty (transgressive segregation) in morphological components and function of the simple and complex jaw systems. Functional novelty was proportional to the prevalence of extreme morphologies in the simple levers; by contrast, recombination of parental morphologies produced transgression in the MTOM 4-bar linkage. We found multiple loci of moderate effect and epistasis controlling jaw phenotypes in both the simple and complex systems, with less phenotypic variance explained by QTL for the 4-bar. Genetic linkage between components of the simple and complex systems partly explains phenotypic correlations and may constrain functional evolution.

Functional phenotypes, such as running speed or jaw bite force, are emergent properties of multiple interacting components and as such exhibit an array of responses to evolutionary pressure (Alfaro et al. 2004). In complex functional systems, manifold morphological configurations can produce similar function. This phenomenon, known as many-to-one mapping (MTOM), is pervasive in biology and has been described as a generality of organismal design (Hulsey et al. 2005; Wainwright 2007). There are several context-dependent implications of MTOM for the generation of emergent function. Because a number of morphologies can produce the same function, structure can evolve without concomitant effects on performance. This may (1) permit individuals to explore morphospace without sacrificing adaptive ancestral function (Alfaro et al. 2004, 2005) and may (2) reduce trade-offs between dual-use components of multiple systems (Holzman et al. 2011). On the other hand, given the nonlinear relationship between morphological and functional variance in MTOM systems, subtle changes in morphology could facilitate large functional shifts (Alfaro et al. 2004; Hulsey et al. 2005; Wainwright 2007). The evolutionary pliability and redundancy of MTOM traits may have played an underappreciated role in the evolution of phenotypic and functional diversity (Alfaro et al. 2005; Parnell et al. 2008; Losos 2011).

The oral jaws of teleost fishes provide an excellent contrast between singular and multiple mapping of form to function within the same craniofacial apparatus (Hulsey et al. 2005). A simple lever characterizes the transmission of motion in the lower oral jaw (known as velocity ratio [VR]). Lower jaw VR is dictated by the lengths of the output and the input components (links) of the lever (out-lever/in-lever, see Fig. 1; Westneat 2003). Higher values of VR characterize jaws capable of more extensive transmission of motion (“faster” jaws), whereas lower values are associated with greater force amplification (“stronger” jaws). In this simple lever system, a single functional value arises from any particular morphological combination (one-to-one mapping, OTOM), and variance in link length scales linearly with variance in VR (Alfaro et al. 2004).

**Figure 1:**
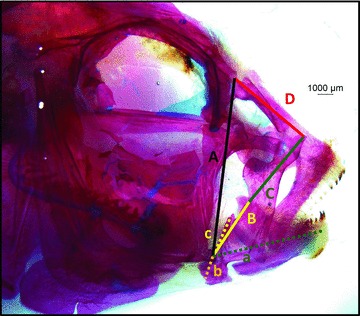
The structural components of the lower jaw levers (dashed) and complex anterior jaw 4-bar linkage (solid) displayed on a cleared and stained cichlid head. The elements of the two simple lower jaw levers are represented with differently colored dashed lines: (a) green = out-lever (LJout); (b) orange = opening in-lever (LJin); (c) yellow = closing in-lever (LJincl). The anterior jaw 4-bar linkage is represented with differently colored solid lines: (A) black = suspensorium (AJFix), (B) yellow = input (AJLJ), (C) green = output (AJMax), (D) red = coupler (AJNas).

Alternatively, the dynamic anterior jaw 4-bar linkage shows more complex mapping of form to function than the simple lower jaw lever system (Westneat 1990, 1994; Muller 1996). Function in the 4-bar is modeled as the rotation of the maxillary link (output) given an input of lower jaw depression, influenced by the overall structural configuration of two additional elements (Fig. 1). Function in this ring-like linkage is quantified in terms of kinematic transmission (KT) by measuring the lengths of the four components and geometrically computing the mechanics of the system (Hulsey and Wainwright 2002). Values of KT are functionally and ecologically relevant in teleost fishes as higher KT is associated with greater jaw protrusion and specialization on evasive prey, and lower values likely enhance force transmission and specialization on more durable, attached prey (Hulsey and Garcia de León 2005). Simulations reveal complex dynamics of the 4-bar linkage in response to selection for specific KT values in reef fishes (Alfaro et al. 2004). The 4-bar system is thus an exemplar of MTOM, with multiple, distinct morphological configurations producing similar KT.

Recent work in cichlid fishes illustrates a second, complementary way in which MTOM of the 4-bar system could contribute to complex evolution of form and function. Parnell and colleagues (2008) modeled the biomechanical outcome of hybridization between species whose 4-bar KT was nearly identical, but whose component links differed. They showed how recombination of parental link lengths could produce transgressive (extreme) segregation of KT, at appreciable frequency, in F_2_ offspring. Such extreme phenotypes, if produced from natural hybridization, would hold implications for both phenotypic and lineage diversification (Rieseberg et al. 1999; Seehausen 2004; Stelkens et al. 2009). Taken together, available data suggest that the distinction between one-to-one and many-to-one mapped traits could engender major differences in how these classes of functions evolve, over both macro- and microevolutionary scales (Alfaro et al. 2004; Albertson and Kocher 2005; Wainwright 2007; Parnell et al. 2008). Missing from this discussion is an understanding of how the genetic architecture of simple OTOM and complex MTOM traits contributes to differences in evolvability.

Albertson and colleagues (2003, 2005) reported a small number of quantitative trait loci (QTL) with large, divergent phenotypic effects, and high overall heritability, for the simple lower jaw levers in an intercross between two Malawi cichlid species. Moreover, they demonstrated strong genetic correlation among links of the levers and showed that differences in *bmp4* expression were sufficient to partly explain these correlations (Albertson et al. 2005). These findings make sense because the links of the simple levers correspond to a small number of bony elements, comprising both anatomical and developmental units. Consistent with expectation, QTL for function (in this case VR) in these OTOM lever systems also tended to colocalize in the genome with QTL for link dimensions (Albertson et al. 2005). Genetic correlation between links of the simple lever systems, as observed by Albertson and colleagues, is important because it supports one of the key assumptions of previous modeling efforts (see e.g., Alfaro et al. 2004) that restricted recombination between genes affecting link lengths.

It is less straightforward to derive predictions about the genetic architecture of the MTOM 4-bar system, which includes upper and lower jaws as well as components of the suspensorium. First, the input (lower jaw), output (maxillary), and nasal links correspond roughly with bone lengths and/or developmental units, but the fixed link does not (see Fig. 1). Second, the redundant relationship between component links and function suggests that the genetic basis of KT may not be a simple integration of QTL for link lengths. Simulations of selection on KT have modeled the 4-bar system with up to 50 loci, near-zero recombination between loci and complete genetic control (Alfaro et al. 2004). Although the latter two assumptions are almost certainly unrealistic, the genetic architecture of variance in 4-bar links and/or KT, and the degree to which this architecture differs from the simple lever system, is unknown.

Here, we examine variance in morphology and predicted function as well as the genetic basis of MTOM and OTOM oral jaw systems in F_2_ hybrids derived from an intercross of two rock-dwelling Lake Malawi (LM) cichlid species. This group of rapidly evolving, closely related fishes exhibits dramatic morphological and functional differences in numerous traits including elements of the anterior jaws, tooth and taste bud number, and body coloration (Ribbink et al. 1983; Streelman et al. 2003b; Fraser et al. 2008; Parnell et al. 2008; Cooper et al. 2010; Parnell and Streelman, unpubl. data). Fertile interspecific hybrids among members of this lineage are easily made in the lab, are known to occur in the lake (Smith and Kornfield 2002; Smith et al. 2003; Streelman et al. 2004; Mims et al. 2010) and might have contributed to evolutionary diversification (∼10^3^ species in 10^6^ years; Seehausen 2004; Albertson and Kocher 2005; Genner and Turner 2005; Won et al. 2005). We compare hybrid trait distributions in both simple and complex jaw systems with those found among parental species and across the LM species flock. By QTL mapping with RAD-tag SNPs, we identify genomic regions controlling anterior jaw traits and examine the genetic basis of novel phenotypes resulting from interspecific hybridization. Our linkage groups represent consensus chromosomes across East African cichlids, allowing us to identify genomic regions shared by multiple traits (craniofacial elements, color patterns, sex determiners) from different analyses (Streelman et al. 2003a; Albertson et al. 2005; Roberts et al. 2009; Ser et al. 2010; Parnell and Streelman, unpubl. data).

We test four specific predictions, derived from previous simulation and measurement (Alfaro et al. 2004; Parnell et al. 2008), about the evolution of complex traits: (1) MTOM in the 4-bar linkage will produce transgressive function in F_2_ hybrids (Parnell et al. 2008). (2) More specifically, transgressive function in hybrids will result from recombination of parental 4-bar link lengths (Parnell et al. 2008), whereas recombination of link lengths is not expected for the simple system (Albertson et al. 2005). (3) The lower jaw (input) and maxilla (output) links will be strong (genetic) determinants of 4-bar KT, and QTL for KT will colocalize with QTL for these lengths (Alfaro et al. 2004; Parnell et al. 2008). (4) Finally, the MTOM 4-bar linkage system will exhibit more complex, diffuse genetic architecture and perhaps lower heritability than the lower jaw lever systems.

## Methods

### PRODUCTION OF A MAPPING POPULATION FROM MTOM SPECIES

Two rock-dwelling (*mbuna*) cichlid species from LM were chosen for interspecific hybridization. The dam represents *Pseudotropheus elongatus*, a herbivorous species in which individuals feed on the substrate. The sire (*Cynotilapia afra* Mara yellow) represents one of the few primarily planktivorous *mbuna* species. We chose these species precisely because they exhibit MTOM in the 4-bar linkage system (Parnell et al. 2008; Table 1). The 4-bar morphologies producing nearly identical function (KT) differ between the species as *P. elongatus* has shorter input and output links (AJLJ and AJMax; Fig. 1; Table 1), whereas *C. afra* has longer lengths of these elements. Relative sizes of these elements are evolutionarily and functionally relevant. The lengths of input and output links are positively correlated with one another across a large dataset of 86 LM species and both elements are correlated with KT, yet exhibit different signs of the correlation (AJLJ-KT, positive; AJMax-KT, negative; Parnell et al. 2008). Parnell and colleagues (2008) used a simple genetic model to simulate F_2_ hybrids from the intercross of *P. elongatus* and *C. afra*, and observed a large proportion of F_2_ with transgressive KT, produced by recombination of parental link lengths. Here, we empirically generate this intercross and compare results to those from our simulations. We note the potential for natural hybridization between these species as they co-occur at numerous sites throughout LM and show little spatial partitioning along depth gradients (Parnell and Streelman 2011).

**Table 1:** Anterior jaw trait values for F_2_ hybrid cichlids (*n* = 351) and parental species (*Cynotilapia afra n* = 5 and *Pseudotropheus elongatus n* = 5). Mean, standard deviation, maximum, and minimum values are included as well as calculated threshold for transgressive (tg) trait values (High/Low tg threshold; see Methods). Data includes measures of simple lever structure (opening in-lever = LJin; out-lever = LJout; closing in-lever = LJincl) and function (closing velocity ratio = Close VR; opening VR = Open VR) as well as complex 4-bar linkage structure (input = AJLJ; output = AJMax; coupler = AJNas; suspensorium = AJFix) and function (kinematic transmission = 4-bar KT).

	Simple lever					4-Bar linkage				
LJin	LJout	LJincl	Close VR	Open VR	AJLJ	AJMax	AJNas	AJFixed	4-bar KT
*Lake Malawi species set*										
average	2.75	11.61	4.40	2.71	4.31	5.12	6.96	7.63	13.10	0.77
standard deviation	0.54	2.62	0.64	0.39	1.08	0.94	1.27	1.15	1.44	0.16
maximum	4.6	19.8	6.5	3.76	8.33	8.4	10.8	11.2	18.4	1.59
minimum	1.7	6.3	3.0	2.03	2.28	3.2	4.5	5.0	10.2	0.41
*Cynotilapia afra*										
average	2.64	10.88	4.76	2.30	4.12	5.34	7.28	5.79	13.14	0.73
standard deviation	0.27	0.99	0.44	0.31	0.22	0.36	0.22	0.59	0.76	0.03
maximum	3.0	11.8	5.3	2.57	4.50	5.9	7.6	6.7	14.0	0.78
minimum	2.3	9.2	4.2	1.84	3.95	4.9	7.0	5.0	12.3	0.69
*Pseudotropheus elongatus*										
average	3.36	9.00	4.01	2.33	2.66	3.50	5.07	5.00	11.05	0.70
standard deviation	0.31	0.68	0.40	0.28	0.35	0.82	1.48	0.62	1.08	0.04
maximum	3.7	10.0	4.4	2.58	3.16	4.7	7.3	5.5	12.4	0.74
minimum	3.0	8.6	3.4	2.07	2.36	2.9	4.1	4.1	9.9	0.64
High tg threshold	4.0	12.9	5.6	2.88	4.56	6.1	7.7	7.0	14.7	0.80
Low tg threshold	2.1	7.6	3.2	1.68	1.96	1.9	2.1	3.7	8.9	0.62
*F_2_*										
average	2.61	9.17	3.55	2.62	3.61	3.96	6.46	5.13	11.88	0.62
standard deviation	0.47	0.58	0.45	0.32	0.58	0.35	0.56	0.63	0.72	0.06
maximum	**4.1**^1^	11.1	5.5	**4.14**^1^	**5.33**^1^	5.1	**8.6**^1^	**8.0**^1^	**15.4**^1^	**0.87**^1^
minimum	**1.6**^2^	**7.5**^2^	**2.0**^2^	1.78	2.13	2.6	4.6	**3.4**^2^	**8.1**^2^	**0.45**^2^
Proportion tg high	0.01	0	0	0.17	0.07	0	0.02	0.01	<0.01	0.01
Proportion tg low	0.13	<0.01	0.21	0	0	0	0	0.01	<0.01	0.50
Fxn:strx tg ratio				0.80	0.48					13.85

Transgressive F_2_ values are noted with bold font.

1 if values range above the thresholds set from the parental data.

1 if values range below the thresholds set from the parental data.

To generate the cross, several *C. afra* males were placed in a 189-L aquarium with *P. elongatus* females at a ratio of 1:2. Within a week the dominant male (and subsequent sire) had established an obvious hierarchy over his conspecifics and had fertilized the eggs of the dam. Fin clips (25 mm^2^ anal fin) were taken from both the dam and sire for RAD-tag library preparation and the pair was kept in a breeding tank with dither fish to diffuse male aggression. Offspring were taken from the dam after hatching, grown in net pens and moved to consecutively larger aquaria as they grew. This pair produced several F_1_ broods that were maintained in 189-L aquaria for two years during that time they produced over 600 F_2_ hybrid offspring. Each F_2_ family was kept segregated from other families and numbered by brood sequence (and individual). Three hundred ninety-seven F_2_ hybrids were euthanized (tricaine methane sulfonate, MS-222, in accordance with established Georgia Institute of Technology IACUC protocols) and processed for DNA samples and oral jaw phenotypes.

### MORPHOMETRICS

All F_2_ fishes were collected and sacrificed upon reaching approximately 75 mm total length (TL). Following sacrifice, fishes were fixed in 10% buffered formalin for one week and then stored in 70% ethanol until processing. Individuals were cleared with trypsin and double-stained using alcian-blue (cartilage) and alizarin-red (bone). This method allows clear visualization of the skeletal components while maintaining the articulations among skeletal elements. Cleared and stained individuals were transferred to glycerin with thymol for measurement and storage.

We measured the physical components of the oral jaw functional systems in F_2_ hybrid offspring, both parents, and three additional individuals of each parental species. All jaw elements (see Fig. 1) were measured to the nearest 0.1 mm using dial calipers, and were defined according to previous studies (Barel 1983; Westneat 1990, 1994; Wainwright et al. 2004). We first quantified three links that comprise the two simple levers of the lower jaw. We measured the output lever (LJout) as the distance from the quadrate–articular joint to the tip of the mandible (Fig. 1a). We quantified the lower jaw opening input lever (LJin) as the distance between the quadrate–articular joint and the insertion of the interoperculomandibular ligament on the articular (Fig. 1b). Additionally, we measured the lower jaw closing input (LJincl) as the distance between the quadrate–articular joint and the insertion point of the adductor mandibulae on the coronoid process (Fig. 1c).

The links of the 4-bar system have been described in detail with their respective measurement landmarks (Westneat 1990, 1994; Wainwright et al. 2004; Fig. 1). The critical elements include the fixed link (AJFix), measured as the distance from the attachment point of the nasal bone and the neurocranium to the quadrate–articular joint (Fig. 1A). This link is assumed to be stationary and anchors the motion of the other three elements. The lower jaw (AJLJ) rotates on the fixed link at the quadrate–articular joint forming the input to the anterior jaw 4-bar system, and is measured from this joint to the ligamentous attachment of the dentary on the maxilla (Fig. 1B). Motion is transmitted from the AJLJ to the maxillary link (AJMax), quantified as the distance from the ligamentous dentary-maxilla attachment to the ligamentous connection of the head of the maxilla on the nasal (Fig. 1C). The AJMax thus serves as the output link in this system. The nasal link (AJNas) stretches from the neurocranium to the maxilla (Fig. 1D) and functions to couple, or transfer motion, between the maxilla and the fixed link.

Because structural components of the anterior jaw are highly correlated with body size (Hulsey and Wainwright 2002), we expressed all physical measurements as a proportion of standard length. Standard length is measured as the distance from the tip of the premaxilla to the anterior tip of the caudal fin. Although F_2_ individuals and members of parental species were similar in adult size, we chose this size correction for consistency, as well as to allow comparisons to other Malawi species that differ considerably in size (Parnell et al. 2008; Hulsey et al. 2010). For simplicity, size-standardized measures were used for all analyses, as the size conversion does not change the calculation of function (i.e., VR and KT are dimensionless). Size-standardized jaw measurements were used to calculate the VR and KT of each individual jaw in the simple lever (opening and closing VR) and complex 4-bar linkage as described elsewhere (Westneat 1994; Hulsey and Garcia de León 2005). Simple lever VR was calculated as the ratio of out-lever to the respective in-lever for opening and closing of the lower jaws (Westneat 1994). The calculation of 4-bar KT followed that of Hulsey and Garcia de León (2005), using a starting angle of 15° and an input angle of 30° relative to the lower jaw. VR and KT are used to estimate the amount of motion transmitted through the jaw systems, whereas simultaneously describing the mechanical advantage (MA) or transmission of force as the reciprocal of VR or KT (Hulsey and Wainwright 2002).

To quantify novel phenotypes produced in this cross, we employed a conservative threshold of transgressive segregation (TS; extreme or novel trait values). Hybrid trait distributions are typically bounded by values expressed in the parents; therefore TS has been described as any trait value outside that of the parental species mean (Rieseberg et al. 1999). Parnell et al. (2008) employed a modified definition in simulation whereby the standard deviation of the hybrid trait distribution set the boundaries for transgressive traits. This value encompassed all of the intraspecific variation found in LM cichlids and was a justifiable proxy, as parental trait distributions were unknown. In the present analysis, we have included both parental species distributions and estimates of intraspecific variation across the LM cichlid species flock to produce a conservative yet realistic definition of transgressive trait values. We use the highest and lowest parental species means ±2 standard deviations for each trait as the threshold values for transgression (e.g., see Table 1).

### DNA EXTRACTION, RAD LIBRARY PREPARATION, AND ILLUMINA SEQUENCING

Genomic DNA was purified from sire and dam fin tissue using the DNeasy Blood & Tissue Kit (Qiagen, Valencia, CA) and RAD-tag libraries were prepared and sequenced from each. A sample of 10 μg DNA from each individual was digested with the restriction endonuclease *SbfI* (recognition sequence CCTGCAGG) and RAD-tag libraries were produced as in Baird et al. (2008). DNA was digested for 60 min at 37°C in a 50 μl reaction with 20 units (U) of *SbfI* (New England Biolabs [NEB]). Samples were heat inactivated for 20 min at 65°C. Two microliter of 100 nM P1 adapter (a modified Solexa^©^ adapter, 2006 Illumina, Inc.[San Diego, CA], all rights reserved) were added, each containing a unique multiplex sequence index (barcode) which is read during the first four nucleotides of Illumina sequence. In addition to the adapters, 1 μl of 10 mM rATP (Promega, Madison, WI), 1 μl 10× NEB Buffer 4, 1.0 μl (1000 U) T4 DNA Ligase (high concentration, Enzymatics, Inc., Beverly, MA), and 5 μl H_2_O were added to the samples and each was incubated at room temperature (RT) for 20 min. Samples were again heat inactivated for 20 min at 65°C, pooled and randomly sheared with a Bioruptor (Diagenode, Denville, NJ) to an average size of 500 bp. Samples were run on a 1.5% agarose (Sigma, St. Louis, MO), 0.5× TBE gel and DNA 300–800 bp was isolated using a MinElute Gel Extraction Kit (Qiagen). End blunting enzymes (Enzymatics, Inc.) were used to polish the ends of the DNA. Samples were purified using a MinElute column (Qiagen) and 15 U of Klenow exo− (Enzymatics) was used to add adenine (Fermentas, Glen Burnie, MD) overhangs on the 3′ end of the DNA at 37°C. After subsequent purification, 1 μl of 10 μM P2 adapter (a divergent-modified Solexa^©^ adapter, 2006 Illumina, Inc., all rights reserved) was ligated to the obtained DNA fragments at 18°C. Samples were again purified and eluted in 50 μl H_2_O. The eluate was quantified using a Qubit fluorimeter and 20 ng of this product was used in a PCR amplification with 20 μl Phusion Master Mix (NEB, Ipswich, MA), 5 μl of 10 μM modified Solexa^©^ Amplification primer mix (2006 Illumina, Inc., all rights reserved) and up to 100 μl H_2_O. Phusion PCR settings followed product guidelines (NEB) for a total of 18 cycles. Again, samples were gel purified and DNA bands from 300–700 bp size range were excised and diluted to 1 nM.

### ILLUMINA SEQUENCING

The pooled RAD libraries corresponding to the cichlid dam and sire were sequenced on an Illumina Genome Analyzer IIx at the University of Oregon High Throughput Sequencing Facility in Eugene, Oregon. Illumina/Solexa protocols were followed for paired-end (2 × 60 bp) sequencing chemistry.

#### RAD longRead assembly

The RAD LongRead protocol uses mate-paired Illumina/Solexa data to assemble DNA sequence adjacent to restriction enzyme cleavage sites in a target genome. Unlike randomized short-insert paired end (SIPE) Illumina libraries, LongRead sequence data are characterized by a common or shared single end (SE) read that is anchored by the restriction enzyme digestion site and a variable paired-end read. Similar strategies have been reported recently as methodology for genome assembly in complex genomes using short-read Illumina/Solexa data (Hiatt et al. 2010).

Internal Floragenex sequence tools and perl scripts were used to process Illumina/Solexa data. First, raw fastq sequence data from each cichlid sample were segregated by the appropriate multiplex identifier. To construct RAD LongRead contigs for SNP detection, data from the *C. afra* F_0_ sire (the sequence quality from this individual was slightly higher than that of the dam) were used to construct a reference assembly. Sequences with more than 5 poor Illumina quality scores (converted phred score of Q10 or lower) were discarded (typically <5% of all data). Remaining reads were then collapsed into RAD sequence clusters that share 100% sequence identity at the SE Illumina read. To maximize efficient assembly of sequences contributed from low-copy, single dose genome positions, we imposed a minimum of 25× and maximum 500× sequence coverage at RAD SE reads. These thresholds have been empirically determined; single loci with coverage under 25× often display short and fragmented contig assemblies due to insufficient sequence coverage whereas loci with greater than 500 identical SE reads are often contributed from high-copy contaminant DNA or can be contributed by dosing from multiple genomic loci (e.g., repetitive class sequences). The variable paired-end sequences for each common SE locus were extracted from these filtered sequences and passed to the Velvet® sequence assembler for contig assembly (Zerbino and Birney 2008). The contig data were further filtered to those with coverage higher than 4× (average 5.7×) and lengths greater than 200 bp (average 301.8 bp).

#### SNP detection and selection of informative markers

Custom Floragenex short-read software using a Needleman–Wunsch algorithm was used to align paired-end Illumina/Solexa sequence reads from both cichlid sire and dam samples to the reference cichlid sequence. SNPs were called with a minimum of 2× coverage (two redundant reads in each parent) totaling 29,064 SNPs in 35,696 contigs. Relative to the estimated number of bases sequenced (∼10.7 Mb), this corresponds to a SNP rate of 0.27%, a value consistent with other studies in LM cichlids (Loh et al. 2008). The complete SNP panel was then screened for those alleles free of polymorphic flanking regions (120-bp window) producing 9763 candidate SNPs for genotyping. From a subset of 1079 SNPs at 6× coverage or higher in each parent, we selected 513 which were free of heterozygosity (alternately fixed SNPs) and contained the SNP near the middle of the sequence. In addition, 10 SNPs from a previous project (Loh et al. 2008) were included for a total of 523 markers. These SNPs were analyzed as potential candidates for genotyping with the Illumina® assay design tool, each generating a score from 0 to 1 (> 0.6 have high probability of success). Markers with a designability score of 1.0 and a final score ≥0.84 were selected from our set, resulting in a final SNP genotyping dataset of 384 markers. In the final marker set, 204 exhibited 6–14 times coverage of each alternative allele and 180 loci were 15–30 times coverage. We Sanger sequenced 30 of these loci and confirmed that SNPs were alternately fixed in the F_0_ parents.

### SNP GENOTYPING IN F_2_ INDIVIDUALS

A total of 382 F_2_ individuals as well as sire and dam were genotyped at 384 SNP markers by the Emory Biomarker Service Center (Emory University). We used the Illumina BeadArray genotyping platform coupled with the GoldenGate assay in which oligonucleotide pool assays (OPAs) are designed specifically to discriminate between alleles at an SNP (Oliphant et al. 2002; Fan et al. 2003). Marker genotypes were examined to evaluate accuracy of initial sequencing during SNP discovery and 12 loci were removed because they did not segregate between the parents. Another 12 markers that contained one heterozygous parental genotype were removed, as well as seven others with low genotyping success in either parental or F_2_ individuals. This left a fully informative set of 353 SNP markers, with nearly complete genotypic data across F_2_ (0.6% missing data).

### GENETIC LINKAGE MAP CONSTRUCTION

A genetic linkage map was produced with SNP marker genotype data using JoinMap® 3.0 software (van Ooijen and Voorrips 2001). The map was created using Kosambi's mapping function, a logarithm of the odds (LOD) threshold of 1.0, recombination threshold of 0.4, a jump threshold of 5.0, and a ripple function with no fixed order of loci. A LOD threshold of 4.0 was used to join 344 loci in 22 linkage groups with a total map size of 1082 cM and average marker distance 3.67 ± 1.71 cM. The linkage map generated from this cross was translated to assemblies of tilapia (an East African river cichlid) and *Metriaclima zebra* (another LM rock-dwelling cichlid) genomes (https://www.broadinstitute.org/ftp/pub/assemblies/fish). This was accomplished by co-BLASTing (Altschul et al. 1990) our SNP-containing sequences and microsatellites from the tilapia linkage map (Lee et al. 2005) against both the tilapia and *Metriaclima* assembly scaffolds. Chromosome designations are thus consistent across East African cichlids (Lee et al. 2005; Albertson et al. 2005; Streelman and Albertson 2006) except for a single small linkage group in our study with no marker match in tilapia (“LM22”).

### QTL MAPPING OF JAW MORPHOLOGY AND FUNCTION

The assembled linkage map was used to determine QTL locations for jaw morphology and mechanics in the F_2_ population using the R/qtl package (Broman and Sen 2009). A total of 315 individual F_2_ were included in the analysis after removal of those with incomplete data for the size-standardized jaw measures. We scanned for single QTL using standard and composite interval mapping (CIM), followed by two-dimensional scans to identify pairwise (epistatic) QTL interactions. Using the results of the previous steps, we built multiple QTL models (MQM) incorporating QTL interactions and phenotypic sex as a covariate. In the MQM process, we used a forward–backward selection algorithm to add and remove QTL based on overall model effects and the effects of individual QTL. Our dataset had nearly complete marker genotypes and thus we used the multiple imputation mapping method (see Broman and Sen 2009). Genotype–phenotype associations are scored using the LOD, which represents the log_10_ likelihood ratio comparing the hypothesis of a QTL at a marker location to the null hypothesis of no QTL (LOD = (*n*/2)log_10_ (RSS_0_/RSS_1_), RSS = residual sum of squares; Broman and Sen 2009). The variance in phenotype is assigned to each significant QTL and reported as percent variance explained (PVE) in the analysis output. The variance accounted for by QTL is a proxy for the heritability of a trait and is calculated as 1 – 10^−(2/*n*)LOD^ (Broman and Sen 2009). Significance thresholds for LOD scores were estimated using 1000 permutations of phenotypes (randomized) relative to genotypes (constrained) to build a distribution of maximum genome-wide LOD scores. From this distribution, the 95th percentile LOD score was calculated to serve as a threshold for significant QTL associations (Broman and Sen 2009). We tested whether QTL for these and other traits (Parnell and Streelman, unpubl. data) clustered in the genome by comparing their distribution to a Poisson expectation (following Albertson et al. 2005).

## Results

**Figure 2:**
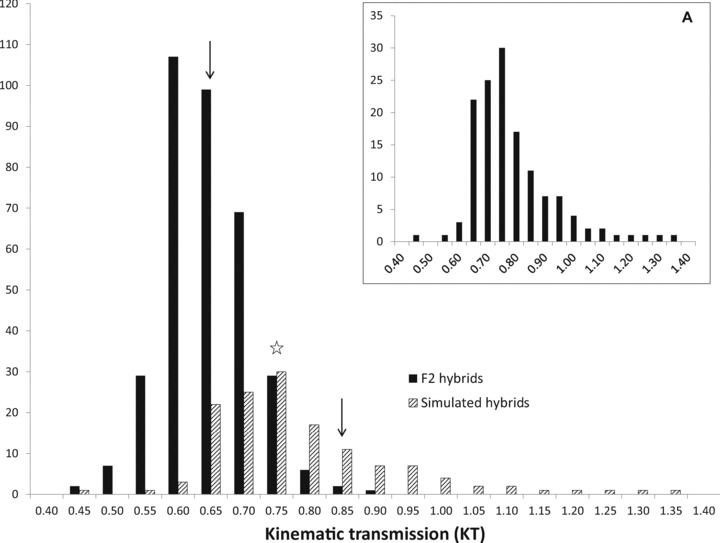
The frequency distribution of 4-bar kinematic transmission (KT) varies widely in Lake Malawi F_2_ hybrids (*Cynotilapia afra*×*Pseudotropheus elongatus*). Both empirical (black bars), and simulated F_2_ hybrids (hashed bars; Parnell et al. 2008) are depicted. The star indicates parental species averages and the two arrows indicate ±2 standard deviations as thresholds for transgressive trait values (see Methods). Inset plot (a) shows distribution of KT in an empirical dataset of 86 Lake Malawi cichlid species (Parnell et al. 2008) on the same axes as the main plot. The range and distribution of the F_2_ approximate the range of KT in the Lake Malawi cichlid flock.

We examined the morphological and functional variance, and the genetic control thereof, in simple jaw lever systems and the complex 4-bar linkage. Oral jaw phenotypes varied substantially in the F_2_ population, with several elements falling far outside of the expectation for intraspecific variation, and in some cases well beyond parental species distributions (Table 1; Fig. 2). A focused look at this variation uncovered unique patterns between structural and functional phenotypes, as well as between simple and complex jaw systems.

### TRANSGRESSIVE PHENOTYPES IN THE ANTERIOR JAW SYSTEMS

Various degrees of transgression were observed in all but one of the anterior jaw traits (Table 1). The size-standardized physical lengths of the simple lever system varied beyond the parental distributions. We observed a small percentage of transgressive out-levers (0.3% LJout), whereas both the opening and closing in-levers (LJin and LJincl) exhibited a greater proportion of extreme values (Table 1). For each in-lever, the majority of lengths were shorter than the parentals (LJin = 12.8% and LJincl = 21.4%), although a few individuals (0.09%) had longer than parental opening in-levers. As one might expect from an OTOM functional system, extreme lever mechanical values (VR) were observed in proportion to the magnitude of transgression in morphology. Both jaw opening VR and closing VR were higher than parentals in 6.6% and 17.4% of F_2_ individuals, respectively, but neither function realized transgressive values lower than the parents (Table 1).

The mean 4-bar KT of the F_2_ (0.62 ± 0.06) was significantly lower than that of the parents (*C. afra*= 0.73 ± 0.03; *P. elongatus*= 0.70 ± 0.04; *t*= 7.23, *P* < 10^−5^) and the F_2_ population showed a much broader range (Table 1; Fig. 2). The distribution of 4-bar KT in the F_2_ spanned large portions of a simulated hybrid dataset as well as empirical data measured across 86 LM species (Fig. 2; Parnell et al. 2008). Over 50% of F_2_ hybrid offspring were transgressive for 4-bar KT, the vast majority of which exhibited lower KT than the parents. By contrast, we observed limited transgression (0–1.7%) in link lengths of the 4-bar system (Table 1). The input link, AJLJ, showed no extreme values and the output link, AJMax, exhibited only 1.7% of values above the parental phenotypes. Similarly, the AJNas and AJFix lengths were transgressive in less than 1% of F_2_ offspring (Table 1). To examine potential effects of morphology on KT, we separated F_2_ individuals into two bins of (1) transgressive KT and (2) not transgressive KT. The input (AJLJ) and output (AJMax) 4-bar links differed significantly in length (AJLJ *t*= 8.4, *P* < 10^−15^; AJMax *t*= 8.1, *P* < 10^−15^) between bins. Finally, we wanted to compare the relationship between morphology and mechanics in terms of the generation of transgressive function (VR and KT). We thus computed a ratio of functional to morphological transgression using data from Table 1. The “transgression ratio” of the 4-bar linkage (13.85) contrasts with that of either simple lever (closing = 0.80 and opening = 0.48; Table 1) and indicates a distinct relationship between transgressive morphology and novel function for each system.

### GENETIC BASIS OF ANTERIOR JAW STRUCTURES AND FUNCTIONS

#### Simple jaw levers

Single and double QTL scans revealed tentative associations for simple lever traits; models with multiple QTLs, covariates, and interactions revealed strong support for several factors and epistatic effects (Table 2). The full model for the opening in-lever (LJin) included eight loci on six chromosomes (15, LM22, 5, 10, 8, 20), plus epistasis, explaining over 40% of the variance (PVE) in this trait with strong statistical support (LOD = 36.59). The genetic basis of the out-lever (LJout) shared by the simple jaw systems was best explained by a model including four QTL (on chromosomes 8, 13, 2, 12) and three interactions (LOD = 24.76, PVE = 30.37). The out-lever was the only simple lever phenotype to exhibit a significant effect of phenotypic sex (longer in females), which accounted for over 5% of the variance in this trait. Four QTL on chromosomes 2, 21, 17, 18 and two interactions were associated with variance in the closing in-lever (LJincl, LOD = 13.64; Table 2).

**Table 2:** Results of multiple QTL mapping (MQM) for factors controlling jaw structure and function in an OTOM system. The simple lever components and velocity ratios (VR) are included as phenotypes. The log-likelihood score (LOD), percent variance explained (PVE), and the *P*-value for the full model are indicated in the top portion. The lower table represents the results of dropping a factor from the model one at a time. Putative QTL are indicated as chromosome@position in centimorgans (cM), “sex” represents inclusion of phenotypic sex as a cofactor, and epistatic interactions are indicated by factor:factor notation. Significant results are indicated as *P < 0.005; **P < 0.0005.

		df	LOD	PVE	*P*	Significance
LJin						
	Full model	40	36.59	41.43	<0.0001	
	Drop one QTL:					
	15@3.7	10	13.90	13.20	<0.0001	
	LM22@0.8	14	11.95	11.18	<0.0001	**
	5@26.6	10	11.61	10.84	<0.0001	
	10@45.0	6	10.57	9.79	<0.0001	**
	8@27.2	6	8.79	8.03	<0.0001	
	10@60.5	6	7.32	6.62	<0.0001	**
	15@14.0	6	6.25	5.60	0.0003	
	20@7.4	6	5.19	4.61	0.0019	*
	5@26.6:10@45.0	4	9.44	8.67	<0.0001	
	5@26.6:10@60.5	4	7.15	6.46	<0.0001	**
	15@3.7:LM22@0.8	4	4.55	4.03	0.0011	
	15@3.7:8@27.2	4	4.11	3.63	0.0024	*
	LM22@0.8:15@14.0	4	4.41	3.90	0.0014	
	LM22@0.8:20@7.4	4	4.34	3.84	0.0016	*
LJout						
	Full model	21	24.76	30.37	<0.0001	**
	Drop one QTL:					
	8@0.0	10	11.03	12.18	<0.0001	**
	13@43.0	6	9.17	9.99	<0.0001	
	2@10.7	10	8.80	9.56	<0.0001	**
	12@49.9	6	5.73	6.08	0.0004	
	sex	1	5.35	5.66	<0.0001	**
	8@0.0:13@43.0	4	7.67	8.26	<0.0001	
	2@10.7:12@49.9	4	3.28	3.42	0.0070	*
	2@10.7:8@0.0	4	3.48	3.63	0.0048	
LJincl						
	Full model	16	13.64	18.07	<0.0001	
	Drop one QTL:					
	2@44.6	6	7.34	9.28	<0.0001	
	21@17.7	6	6.84	8.61	<0.0001	**
	17@38.7	6	5.95	7.45	0.0002	
	18@14.0	6	5.91	7.39	0.0002	**
	2@44.6:21@17.7	4	6.45	8.10	<0.0001	
	17@38.7:18@14.0	4	5.27	6.56	0.0001	**
Close VR						
	Full model	8	6.88	9.57	0.0001	**
	Drop one QTL:					
	2@32.7	6	6.78	9.43	<0.0001	**
	17@7.9	6	5.69	7.85	0.0003	
	2@32.7:17@7.9	4	5.49	7.56	0.0001	**
Open VR						
	Full model	8	8.02	11.07	<0.0001	**
	Drop one QTL:					
	20@7.4	6	7.45	10.23	<0.0001	**
	LM22@0.8	6	6.45	8.79	0.0001	
	20@7.4:LM22@0.8	4	6.05	8.23	<0.0001	**

QTL for the functional parameter of jaw closing (VR) shared a chromosome (2) with the out-lever and the closing in-lever, but best-fit loci were 10–20 cM distant. A second locus for closing VR on linkage group 17 was likewise located on a chromosome containing a QTL (∼20 cM away) for the closing in-lever (LJincl). The model for jaw opening VR included two espistatic loci (on chromosomes 20, LM22) identified in the analysis of the opening in-lever (above). Using PVE from the full model as a proxy of heritability (Broman and Sen 2009), we see that simple lever links have higher heritability than VRs in this cross. Additionally, opening and closing VRs share loci and chromosomes in common with in-levers to a greater degree than the common out-lever (Table 2). In contrast to previous study of jaw levers in different species of Malawi rock-dwelling cichlids (Albertson et al. 2003, 2005), we do not detect strong genetic linkage (e.g., common QTL) among component links.

#### Complex 4-bar linkage

MQM for each 4-bar link as well as KT provided support for genetic effects, epistasis, and the role of phenotypic sex in controlling the variance of this functional system (Table 3). Genetic loci associated with variance in the input link (AJLJ) included a marker on chromosome 7, near an XY sex determiner (Parnell and Streelman, unpubl. data) and a marker on chromosome 18 (at position 14 cM), coinciding with the closing in-lever (above). The final model included an epistasic interaction between these two loci and showed moderate heritability (LOD = 4.93, PVE = 6.96). Variance in the output link of the 4-bar system (AJMax) was associated with two interacting QTL (LOD = 7.35, PVE = 10.19), including a locus on chromosome 8 (at position 27 cM) mapped coincidently with the opening in-lever (Table 2). The best-fit model for the coupler link (AJNas) included two interacting QTL (LOD = 6.23), accounting for an estimated 8.71% of the phenotypic variance in this trait. This link shares a chromosome with the input link (AJLJ, chromosome 7) at a position 4 cM proximal to the former. We did not detect QTL for the suspensorium link (AJFix) of the 4-bar system. However, there was a significant effect of phenotypic sex (explaining 6.41% of PVE) on the fixed link, whereby female fixed links were longer.

**Table 3:** Results of multiple QTL mapping (MQM) for factors controlling jaw structure and function in a MTOM system. The 4-bar components and kinematic transmissions (KT) are included as phenotypes. The log-likelihood score (LOD), percent variance explained (PVE), and the *P*-value for the full model are indicated in the top portion. The lower table represents the results of dropping a factor from the model one at a time. Putative QTL are indicated as chromosome@position in centimorgans (cM), “sex” represents inclusion of phenotypic sex as a cofactor, and epistatic interactions are indicated by factor:factor notation. Significant results are indicated as *P < 0.05; **P < 0.005; ***P < 0.0005.

		df	LOD	PVE	*P*	Significance
AJLJ						
	Full model	8	4.93	6.95	0.0045	
	Drop one QTL:					
	7@35.8	6	4.52	6.36	0.0024	
	18@14.0	6	3.72	5.21	0.0102	*
	7@35.8:18@14.0	4	3.33	4.65	0.0048	
AJMax						
	Full model	8	7.35	10.19	<0.0001	
	Drop one QTL:					
	19@53.3	6	5.24	7.15	0.0006	
	8@27.2	6	4.70	6.39	0.0017	***
	8@27.2:19@53.3	4	2.64	3.53	0.0187	
AJNas						
	Full model	8	6.23	8.71	0.0004	
	Drop one QTL:					
	14@54.3	6	5.92	8.25	0.0002	
	7@31.8	6	5.71	7.95	0.0003	***
	14@54.3:7@31.8	4	5.34	7.41	0.0001	
AJFix						
	sex	1	4.53	6.41	<0.0001	
4-bar KT						
	Full model	8	6.03	8.44	0.0006	
	Drop one QTL:					
	8@27.2	6	6.01	8.41	0.0001	
	13@0.0	6	5.62	7.84	0.0003	***
	13@0.0:8@27.2	4	5.60	7.81	<0.0001	

**Figure 3:**
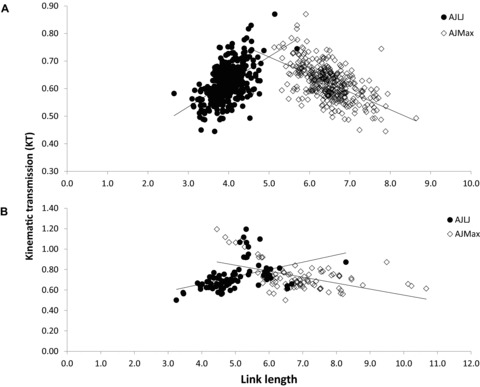
The input (AJLJ) and output (AJMax) links of the 4-bar are correlated with kinematic transmission (KT). Plot (a) represents F_2_ hybrid data (AJLJ r = 0.52, AJMax r =–0.57) and (b) represents empirical data across the Lake Malawi flock (AJLJ r = 0.41, AJMax r =–0.52; Parnell et al. 2008).

Variance in KT of the 4-bar system was associated with two QTL exhibiting epistasis, accounting for 8.44% of the variance (LOD = 6.03; Table 3). One of these QTL was found on a chromosome not detected in analyses of any of the constituent 4-bar links (13@0.0). The largest effect QTL for this functional trait mapped coincidently with a locus explaining variance in the output link (AJMax; 8@27.2). Overall, the 4-bar system exhibits less heritability in this cross than the simple lever systems, and 4-bar KT is phenotypically (Parnell et al. 2008; Fig. 3) and genetically associated with the maxilla (output) link (Table 3).

## Discussion

The relationship between form and function occupies a central position in evolutionary thinking (Wainwright 2007), yet parsing the code from genes to morphology to performance has been difficult (Arnold 1983). Recent theory suggests that systems exhibiting a simple mapping between morphology and function may follow distinct evolutionary dynamics from systems in which morphology relates to function in a complex way (Alfaro et al. 2004). In this study, we simultaneously investigated the phenotypic and genetic variance associated with simple and complex biomechanical systems within the craniofacial apparatus of LM cichlid fishes. We demonstrated that both simple, one-to-one mapped, and more complex, many-to-one mapped traits can exhibit transgressive phenotypes following hybridization. However, these systems differ with respect to the proportion of underlying structural novelty associated with functional transgression. In the simple lever system, transgressive function is associated with greater frequencies of transgressive morphology. By contrast, extreme function is produced in the near absence of extreme morphology in the complex, MTOM 4-bar linkage system.

### TRANSGRESSION IN THE SIMPLE LEVER SYSTEMS

The simple levers consist of only two interacting components and have been shown in simulation to exhibit morphological evolution that is precisely tracked by mechanical evolution (Alfaro et al. 2004). As such, if novel jaw lever function (transgressive segregation in VRs) was exhibited among the F_2_, we expected novel functions in proportion to the degree of transgressive link morphology. True to expectation, transgression observed in lower jaw opening and closing VR was directly related to extreme in-lever lengths in the F_2_ population (Table 1). Transgressive structural links of opening and closing in-levers were nearly always shorter than those found in the parents, and all transgressive VR values were thus higher than parental bounds (i.e., >VR = similar out-lever/shorter in-lever). For instance, more F_2_ individuals exhibited extreme closing in-levers and thus transgressive closing VR. Therefore in this hybrid cross, a major factor facilitating (and/or constraining) transgressive function (and the direction of transgression) for the simple jaw lever systems is the occurrence of transgressive morphology.

Previous analysis of a different intercross between Malawi rock-dwelling cichlids suggested that transgression in the simple system was constrained by a history of strong directional selection and genetic linkage among components (Albertson et al. 2003, 2005; Albertson and Kocher 2005). In this sense, our observation of extreme values in morphology (and mechanics) of the simple jaw levers was unexpected, and thus it is useful to compare and contrast our analysis with that of Albertson and colleagues. They crossed a generalized feeder (*M. zebra*) with a square-jawed, obligate algae scraper (*Labeotropheus fuelleborni*), and identified QTL for lower jaw lever links and VR on cichlid chromosomes 1, 6, 7, 12, 19, and 22, including a signal on chromosome 19 which may correspond to the gene *bmp4* (Albertson et al. 2005) and a QTL on chromosome 12 which may correspond to *ptc*, a receptor in the Hedgehog pathway (Loh et al. 2008; Roberts et al. 2011). Notably, our cross between a generalized algae eater (*P. elongatus*) and a planktivore (*C. afra*) uncovered only a single chromosome in common (chromosome 12, Table 2), but for a different trait.

We did not detect genetic correlations among link lengths, as did previous analyses (Albertson et al. 2003, 2005). Moreover, our study was powered to detect epistasis and this was a key component of the genetic architecture for the simple levers. Thus, we did not observe the signature of consistent directional selection on additive QTL for elements of the lever systems. We note that recombination alone is theoretically insufficient to generate large numbers of transgressive F_2_ for the simple levers in this cross (i.e., swapping the shortest and longest [or vice versa] F_0_ in- and out-levers would not result in transgressive VR), given our definition of the phenomenon. Taken together, the data suggest that the lower jaw lever systems have distinct genetic architectures in the two crosses and that there are many alleles, with different evolutionary histories, affecting these functional systems in Malawi rock-dwelling cichlids.

### MTOM PRODUCES EMERGENT FUNCTIONAL DIVERSITY

The 4-bar linkage system incorporates twice the number of interacting morphological components (four vs. two) of the jaw levers and exhibits a complex relationship between form and function characterized by MTOM (Alfaro et al. 2004; Parnell et al. 2008). The fact that our parental species are exemplars of MTOM in 4-bar function (i.e., nearly identical KT from different morphologies), coupled with the expectation of genetic recombination among loci controlling component links (Parnell et al. 2008), led to the prediction of transgressive 4-bar function in this hybrid cross. As predicted, 4-bar KT ranged widely beyond the distributions seen in either of the parental species. In fact, the F_2_ KT distribution (1) extends beyond that found throughout the rock-dwelling clade (*mbuna* KT range = 0.58–0.85; Parnell et al. 2008), (2) spans a significant portion of the entire Malawi species range (Fig. 2), and (3) approaches the range observed across entire families of reef fishes (Westneat 1995; Wainwright et al. 2004; Alfaro et al. 2005; Cooper and Westneat 2009). This result was not observed for simple lever mechanics whereby opening and closing VR were confined to a narrow portion of the LM distribution (Hulsey et al. 2010).

Transgressive KT was produced in the 4-bar linkage in the near-absence of extreme morphology (Table 1). Less than 4% of all 4-bar link lengths were outside the parental distributions, yet over 51% of individuals exhibited transgressive 4-bar function. Novelty in function without corresponding extreme differences of link lengths indicates that recombination of 4-bar system components is the genetic mechanism responsible for transgressive KT values in this cross (Parnell et al. 2008). These empirical results, contrasted with the simple lever systems, illustrate the effect of component redundancy and the decoupling of morphology and mechanics inherent in MTOM systems (Alfaro et al. 2004). Our analysis of jaw levers and the 4-bar linkage in the same intercross suggests that both the interplay of loci in the genome (epistasis) and the genetic correlation of links between functional systems may underlie cichlid trophic divergence.

### CORRELATION AND CONSTRAINT WITHIN AND BETWEEN FUNCTIONAL SYSTEMS

Our quantitative genetics framework allows us to examine phenotypic correlations within and between simple and complex functional systems, as well as the genetic linkages that may contribute to correlation. For example, empirical and simulation data demonstrate that both the input (AJLJ) and output (AJMax) components of the 4-bar system strongly influence KT (Alfaro et al. 2004; Parnell et al. 2008). Among the F_2_ (and across the entire LM flock), these links are positively correlated with each other (*r*= 0.38) yet exhibit an opposing relationship with respect to 4-bar function (AJLJ-KT *r*= 0.52, AJMax-KT *r*=−0.57; Fig. 3). Such association is at least partly due to genetic correlation between structure and function. Four-bar KT maps to QTL on two chromosomes, and one of those is precisely colocalized with a locus controlling the length of the 4-bar output link (AJMax; 8@27.2, Table 3). The phenotypic correlation between the input and output links is probably not due to genes (we detected no shared QTL), but the relationship may be driven by a functional and/or developmental constraint maintaining proportionality of upper and lower jaws. Notably, the QTL on chromosome 8 (@27.2), influencing maxilla length and 4-bar KT, is also associated with the length of the lower jaw opening in-lever (Table 2).

We detected epistasis and/or a significant effect of phenotypic sex on every morphological and functional trait in this analysis (Tables 2 and 3). For both links affected by sex (lower jaw out-lever and the 4-bar fixed link, Fig. 1), females exhibited longer elements, perhaps as a response to their unique role as maternal mouthbrooders wherein greater buccal volume and higher velocity transmission when gathering young are presumably advantageous. The evolutionary role of such sex–gene and gene–gene interactions is the subject of theoretical debate (Phillips 2008), but our empirical data imply that these effects may be common, and that future studies should be powered to detect them.

Our QTL analysis shows how correlations among elements might create functional conflict between simple and complex systems. To illustrate this point, we note that the simple and complex jaw systems do not share exact component structures, but some of the links and levers are morphologically related (see Fig. 1). The length of the closing in-lever (LJincl) is strongly correlated across F_2_ with that of the 4-bar input (AJLJ) link (*r*= 0.83). Moreover, these lengths share a coincident QTL, located on chromosome 18 (@14 cM, Tables 2 and 3). It is straightforward to see how increasing the length of the closing in-lever will reduce jaw-closing VR and increase jaw-closing force (Albertson et al. 2005). An opposite effect is predicted for the 4-bar system; increasing the length of the input link is correlated with higher KT (Fig. 3; Parnell et al. 2008). This (genetic) correlation between elements of the two systems may impose a constraint such that it is difficult to simultaneously optimize *mbuna* oral jaws for both high lever VR and high 4-bar KT. Notably, these functional values are inversely related in the F_2_ (*r*=−0.32), and the distribution of KT is biased toward lower values (Fig. 2; Table 1). Our analysis suggests that conflict between systems could be mitigated in at least two ways. All else being equal, the greater heritability for simple rather than complex jaw systems (Tables 2 and 3) indicates that the levers will exhibit a more rapid and predictable response to selection. Second, the precise attribute of complexity (MTOM) in the 4-bar system may serve to dampen functional trade-offs (Wainwright 2007; Holzman et al. 2011), if other link combinations evolve. It is clear for instance that members of the sand-dwelling sister clade to the *mbuna* have evolved both high KT and high VR (Parnell et al. 2008; Hulsey et al. 2010).

### GENETIC ARCHITECTURE, TRAIT LINKAGE, AND MODELS OF DIVERSIFICATION

**Figure 4:**
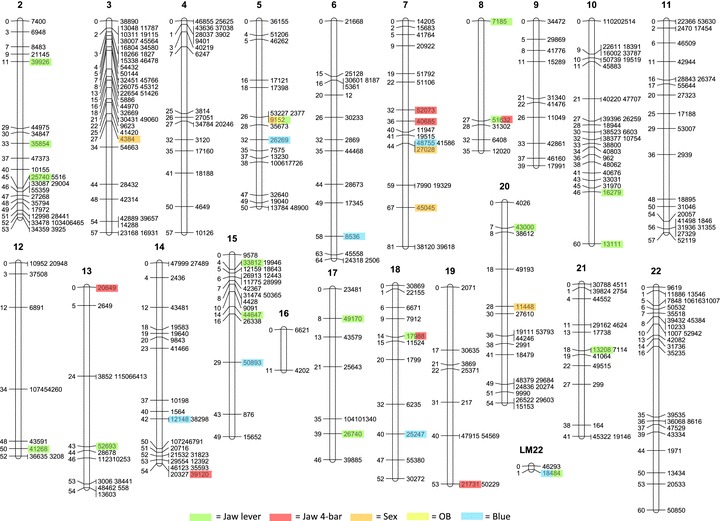
RAD-tag SNP genetic linkage map generated from this Lake Malawi intercross. Chromosome labels are consistent across East African cichlids. Markers are single nucleotide polymorphisms (SNPs) with relative distance in centimorgans (cM). Colored bars represent QTL detected for various traits mapped in this F_2_ population. Simple jaw levers = green; complex 4-bar linkage = red; sex = orange; orange-blotch color = yellow; blue color = blue. For sex, orange-blotch and blue color, see Parnell and Streelman (unpubl. data).

Rapid evolutionary diversification might be facilitated when selection acts on genomic regions controlling multiple functional traits (Peichel et al. 2001), or functional traits linked to markers of reproductive isolation (Hawthorne and Via 2001; Streelman et al. 2003a). We identified large- to moderate-effect loci associated with adaptive male and female coloration as well as multiple sex determination systems (XY and ZW) in this cross (Parnell and Streelman, unpubl. data). Twelve of 13 QTL detected in that analysis are clustered on chromosomes with QTL affecting anterior jaw structure and function (*P* < 10^−7^ under Poisson distribution; Fig. 4). Genomic intervals controlling putatively adaptive functional traits are thus colocalized with those influencing sex determination, as well as marker traits implicated in sexual selection and sexual conflict (Roberts et al. 2009). Theory implies that lineage splitting may be more rapid and more likely when loci controlling ecologically relevant traits and those affecting mating preferences become linked (Gavrilets 2003). The reduced recombination around areas of sex-determining loci (Bull 1983; Fisher 1931; Rice 1987) or inversions (Kirkpatrick and Barton 2006) could facilitate such linkage, making such areas prime genomic sites or “hotspots” for correlated selection (Counterman et al. 2010; Joron et al. 2011). The location of QTL for simple and complex jaw function, plus male- and female-biased color patterns, on the major XY and WZ sex chromosomes of Malawi cichlids (chromosomes 5 and 7) is consistent with such theory. Within models of adaptive evolution and hybrid swarms, it is apparent that the clustering of loci, as detected here, may partly explain parallel bursts of evolution seen in this and other adaptive radiations (Dieckmann and Doebeli 1999; Kondrashov and Kondrashov 1999; Streelman and Danley 2003; Seehausen 2004).
